# American Academy of Dental Science

**Published:** 1897-07

**Authors:** 

**Affiliations:** American Academy Dental Science


### Reports of Society Meetings.

### AMERICAN ACADEMY OF DENTAL SCIENCE.

The regular monthly meeting of the American Academy of Dental Science was held at Young's Hotel, Boston, January 6, at six o'clock P.M., President Andrews in the chair. The paper for the evening was read by Dr. J. L. Hildreth, of Cambridge, Mass. Subject: "To what Extent is Typhoid Fever Preventable?"

*President Andrews.*—Fellows of the Academy, it was my privilege last evening to attend the meeting of the New York Institute of Stomatology. Many of its members are also members of the Academy. Among them I met Drs. Benj. Lord, Geo. S. Allan, and E. A. Bogue, who wished me to remember them to the fellows of the Academy.

I have the honor to-night to present to the Academy Dr. John L. Hildreth, of Cambridge, who will read the paper for the evening.

(For Dr. Hildreth's paper, see page 373.)

### DISCUSSION.

*Dr. Smith.*—Dr. Hildreth has very charmingly taken us from the mouth and the teeth to the intestinal tract. I would like to ask Dr. Hildreth if there is any good reason for a competent physician mistaking the diagnosis in typhoid fever? I know of a couple of cases where such a mistake was made. The patients were travelling abroad and were taken ill. They were supposed to have thoroughly competent physicians, and were treated some time before typhoid fever was ever mistrusted. It was not positively known until another physician was called and diagnosed the case as typhoid fever. From the facts which Dr. Hildreth has so ably put to us to-night we learn that typhoid fever is transmitted only through the excreta of patients who have that disease, and while he stated that the bacillus which produces typhoid fever bears a close resemblance to a harmless bacillus which is common in the intestinal tract, I believe he stated that it can be distinguished. Now, if that be true, why is it not possible, with proper care, to diagnose every case of typhoid fever as typhoid fever at once, without making an error which may lead to the loss of one or more lives? The doctor also remarked, in speaking of that case, of the son of the milkman, along whose route the epidemic had prevailed, that it was somewhat of a question whether the young man had typhoid fever, but the suspicion was confirmed at the autopsy. Now, my point is, whether the presence of the disease could not have been detected before the young man's death?

Another question occurred to me during the course of his remarks, and that is, What becomes of the bacillus on the patient's return to health? We understand that after the bacillus has been introduced into the system, it finds a favorable habitation in the intestines, where it begins to produce its toxin; the system immediately rebels and sets about to manufacture an antitoxin,—in other words, there is a war going on between the system and its antitoxin and the bacillus and its toxin. I suppose that war goes on until the bacillus is entirely stamped out and expelled from the system.

Still another question I would like to ask, How many of us are using cooked milk? I drink a great deal of water, and I feel quite secure in that respect, because I use "Poland Spring" water; but as to milk, I use it on my oatmeal or cracked wheat in the morning, and now and then take a glass of milk at my meals, but as yet have paid no attention to its cooking or sterilization. Of course, I have been aware of its danger, but not so intensely aware as I am at this moment, and I would like to ask Dr. Hildreth and others if they use that precaution in their own households?

*Dr. Hildreth.*—The last question which the gentleman has asked is the easiest, so I shall answer that first by saying that I am one of those extravagant persons who have their own cows, so I do not have to give myself much concern about the milk that we use; but there have been times when I have not manufactured my own milk; and when such occasions arise, I sometimes have bought Pasteurized milk, and I have sometimes Pasteurized it myself, sometimes have it boiled, but never use it as we buy it from the dairy or grocery. The danger in some cases is very small and in other cases very large; we do not know where the danger lies, and I think there is some risk all the time. If you want to be on the safe side, the best way is to get into the habit of cooking your milk the same way as other articles of food. Sometimes, when a man is travelling, he will say to himself as he comes to one of those "ten minutes for refreshments" stations, "I will get off and get a glass of milk and a sandwich." Now, I would not drink a glass of milk in a railroad station for five dollars, and yet I am past the age at which there is an ordinary likelihood of my contracting typhoid. When I am in the country I never care to drink a glass of milk unless I know about it. My attention was first strongly directed to this by a remark of Professor Sedgwick, who said that he could not be induced to drink a glass of milk away from home unless it was guaranteed by some physician, or he knew it had been properly sterilized. Pardon me if I say a word right here about cooked milk. It is not quite as digestible as the uncooked. Milk that is fresh, within a few hours after having been drawn, is very digestible; milk that has been pasteurized or boiled is not quite so digestible, and that is something which has to be borne in mind.

The other question which the gentleman asked, that of diagnosing typhoid fever, was something that I should like to have alluded to, if I had not feared that I should have wearied your patience; but with your permission I will now refer briefly to it.

The question of making an early diagnosis is one of those very interesting problems where all the information we can obtain is not always sufficient to enable us to make a decision at once. Our first knowledge of the case is that the patient has been suffering for five or six days from malaise, headache, diarrhœa, weariness, some little fever and restlessness at night, and these prodromata may continue for a period of fourteen days, and then generally comes a chill, and that is the time at which you generally date the beginning of the disease. Now, when the chill comes on, if it be a case of typhoid, you will find the temperature higher in the evening and a little lower in the morning in a perfectly regular way, and you will have a slight distention of the bowels, with frequent discharges which are yellow, like the dejections from infants. And yet, we may have all these symptoms, and not be able to say that it is a case of typhoid. In cases where there is such doubt, I have treated them just as though they were typhoid. I have observed all the precautions with regard to the destruction of the excreta, and have warned the families in such cases that great care should be taken to prevent the possibility of its spreading. Now, the question is, Why cannot the physician in the early part of this disease, during the first week, when the diarrhœa is pretty well pronounced, when it contains more of the bacilli than in the later stages,—why cannot he take the microscope and find out if the typhoid bacillus is there? If he found them he would say right off, You have got typhoid fever. Unfortunately, the making of the examinations of the stools, of the excreta, is somewhat difficult, and in hospitals, where they have the best facilities for that kind of work, it is not often done; they prefer to keep the patient under proper care and wait a few days for developments. Now, when the fever gets on to about the eighth day, you usually get rose-spots upon the abdomen; then you may be secure in your diagnosis. But suppose you have a case where the rose-spots do not appear? Then you think of acute tuberculosis, galloping consumption. You very often have a cough in typhoid fever, and this, with the fever and emaciated appearance of the patient, might lead you to think, This may be a case of acute tuberculosis.

With reference to the Somerville case, I did not intend to say that the physician in charge had any doubt as to its being typhoid; it was the patient's father, who insisted that his son did not have typhoid, but an autopsy was held which proved the physician was right.

An important discovery in regard to the serum of the blood of typhoid patients has been made by Widall, which now aids us wonderfully in diagnosing the disease. If you take a patient who has the disease sufficiently pronounced, so that his system begins to manufacture an antitoxin, to oppose the toxins of the typhoid bacillus, and prick his ear and get a few drops of blood (Widall, in his first experiments, drew thirty or forty drops), and put it under a little glass and allow it to stand a while, it will separate into the serum and the clot; then take some of this serum and introduce into it a pure culture of typhoid bacilli, which I have told you were moving actively in all directions. If you observe this culture under a glass of the twelfth immersion, when this serum comes into contact with the bacilli they at once show that they have met with something they do not like. In three or four minutes they all huddle together as if they were trying to get away from something, and in ten or fifteen minutes they all remain perfectly still, as if they were overcome. The effect of this experiment is to show us that the serum that we took from the ear of the patient who was supposed to have typhoid was really manufacturing antitoxin, and that the patient who was supposed to have typhoid did really have typhoid. I have applied this test to almost every case that I have had at the hospital this fall, and with the best of success. The question comes, How early can we use this test, how soon does the antitoxin of the system become powerful enough to effect the bacilli of a culture? I think there is a doctor in one of the New York hospitals who claims that this effect can be obtained as early as the fourth day. You see this test may be very valuable when there is a patient in the ward about which there is a doubt. He has advanced, say, to the eighth day, and he has not had any rose-spots, and you are in doubt whether he is developing miliary tuberculosis, typhoid, or some similar disease. You draw a few drops of his blood, allow the serum to separate from the clot, and put the serum into a pure culture of the typhoid bacilli, and the very active germs, which are roaming in all directions, in the course of five or eight minutes are all moving together, and a few minutes afterwards there is not a sign of life in the whole of them. Now, how much this test will be perfected and to our knowledge of how best to oppose the toxin made by this bacillus it is difficult to predict. As I have stated before, we are not altogether hopeful of evolving a system of serum therapy which may effect the typhoid bacillus in a manner similar to that in which antitoxin acts on the diphtheritic bacillus. The effect of the serum of the typhoid fever patient upon the bacillus in its pure culture shows that if we could artificially produce this antitoxin we would be in as favorable a position with regard to the treatment of typhoid as we are now in the treatment of diphtheria.

*Dr. Brackett.*—I wish to express my admiration for that to which we have listened this evening. I have been glad to note the practicality of all that has been put before us, and happily surprised that the speaker should tell us that immunity from the risk of taking disease from milk is so readily provided. I had supposed that brief boiling was not sufficient to guarantee destruction of typhoid fever germs. The matter of conveying disease through milk was brought to my attention several years ago by a lady who had a young son a student in an English school, and she said it was the custom of that school to boil the milk before using. In our household we have long practised scalding all the milk, and for us it is made more palatable by the process. Our water-supply is rain-water, carefully gathered and stored, and we do not drink much of that without cooking.

I would like to ask whether it is supposed that in contaminated streams or water-supplies, with the water at ordinary temperatures at any season of the year, there goes on proliferation of typhoid germs, and what is known concerning their reproduction outside of the human body.

*Dr. Hildreth.*—It is not altogether clear to biologists whether they do propagate in large quantities or not. How and where they propagate outside of the body has never been discovered. You can grow them in some media, but on account of the great activity of the germ no one has ever seen the process of reproduction, or knows how or when it takes place.

*Dr. Briggs.*—I would like to ask one question in regard to the original contamination of the milk-supply,—whether Dr. Hildreth thinks it is due to the cow drinking contaminated water, or to the handling of cows' udders by men who have not taken care of their hands, or possibly to the water which may have been added to the milk, or to the water which may have been used in rinsing out the cans?

*Dr. Hildreth.*—The majority of cases are undoubtedly due to the hands of men who do the milking or who handle the milk. It is possible for a cow to transmit it simply from drinking contaminated water. The number of cases would probably be small that could be traced to the rinsing out of the cans or the addition of water to the milk.

*Dr. Briggs.*—Then, in other words, if the hands of the men who work about the farm, and who handle the milk, are clean, and there is no water added, you would expect no danger from that milk?

*Dr. Hildreth.*—Not from typhoid.

*Dr. Williams*—I would like to ask Dr. Hildreth if observations or investigations have been made the results of which would lead one who has had typhoid fever to hope that he has become immune from typhoid hereafter?

*Dr. Hildreth.*—The fact that typhoid fever is so rare the second time shows that a person who has recovered from an attack of it may expect a reasonable degree of immunity. In that respect it is different from diphtheria. A person may recover from diphtheria and the immunity not remain more than two or three months, and in most cases it does not remain longer than a year or two. This has been proved by the examination of the serum of patients who have recovered from diphtheria; it has been observed that the antitoxin of the system disappears after a little while, and the patient is again susceptible to the disease. The immunity is much greater in typhoid, but unfortunately is not complete. I have known of a case where a patient succumbed to the third attack of typhoid fever.

*Dr. Clapp.*—I would like to ask Dr. Hildreth if the Pasteur filter or the Boston filter excludes the bacilli of typhoid? I have a personal interest in asking this question. For the last six years I have used in my family water of the Boston supply, but it has all been filtered through a Pasteur filter or a Boston filter, and I have used the latter for a large portion of the time. It is attached to the pressure and connected with a glass globe that holds about two gallons. It is arranged automatically, so that when a certain amount of water enters the globe the supply is cut off. Now, we sometimes use that globe for perhaps three months without cleaning it out, and at the end of that time there is not enough sediment in the globe to soil a white handkerchief if used to wipe it out.

The reason that I have used water in this way in preference to the celebrated springs of Boston and vicinity is that to my mind I am surer of obtaining pure water, and I can tell you a little story which I think you will agree fully justifies my belief. A gentleman that I know was very much enamoured of a certain well-known spring water; no other water would do, and he used so much of it that he had it come by the barrel, and after using a portion of one of the barrels he thought he noticed something wrong about it, and an investigation showed a mouse in the barrel. A mouse will not go through my filter.

*Dr. Hildreth.*—With reference to the Boston filter, I cannot speak very certainly. It seems to me that it should be a good filter. The other day my attention was called to it, and this same question was asked me, and, while I have never used one, I have examined it carefully; had the cover taken off and looked all through it, and it seems to me it should be a good filter, and that the water that passed through it should be sterile.

With reference to the Pasteur filter, when they were first introduced, scientific men were very much interested to see if the water, after passing through it, was really sterile water. Many experiments were made abroad, and at the Institute of Technology, with their large and completely equipped laboratories, they made a series of investigations and found that the number of bacteria in the filtered water was very small; the most they found in any of those experiments was two-tenths of one per cent., which is practically pure water. I have used one of the Pasteur filters for some time in my house. The president of the State Board of Health, Dr. Wolcott, has given his attention very largely to this matter of pure drinking water, and he is a better authority on the subject than any one that I know, and it is reported that he says he would be perfectly willing to drink the water of Chicago, which Professor Sedgwick had examined and found to contain several different kinds of dangerous germs, if it only went through a Pasteur filter. I think a Pasteur filter is absolutely germ-proof.

*Dr. Barker.*—Speculation is sometimes interesting if it is not profitable. We have heard several things this evening which might make good subjects for speculation, and one of them is, Where is the native heath of this species of life which we have heard so much about? We know that the home of the elephant is in Asia, the home of the lion in Africa, of the kangaroo in Australia, etc., but where is the home of the typhoid bacillus? Where do they come from? How do they get into the water or the milk which we drink? Do they grow there spontaneously? Scientists tell us that such a thing is not possible. The same power that made a bacillus made a banyan; the same power that made a micrococcus made the moon. You know it is said that "great fleas have little fleas upon their backs to bite 'em, and these again have lesser ones, and so on, *ad infinitum.*" We must get water into the system and we must take food. The doctor has told us that if we wished to be sure of escaping germs we must not drink milk or surface water unless it has been boiled. Now, it is impossible for most places to get water from subterranean sources by vast systems of wells, driven so far below the earth's surface as to be below contamination. That is one thing that I think our municipalities and boards of health are remiss in, not directing attention to the advantages of subterranean sources of water. How manifestly impossible it is to get pure water from the vast water-sheds that feed our water-supplies! We find that cattle, horses, and sheep graze and live upon the hill-sides, and when there comes a rain everything on these hill-sides is washed into the rivers and brooks which so many people are obliged to use for drinking water. It is a comfort to know that if we are obliged to drink surface water that we may escape danger by boiling the water, but above all and beyond all, and it is a point I think is too often lost sight of, we should aim to keep ourselves in such a condition of health that these germs will have no effect on us. It seems to be conceded that the man who is a vital man, who is brim-full of light and health, enjoys immunity from their attacks. That is one way, and that, in my opinion, is the better way for us to combat them. Lice, as we know, attack weak, puny plants, they attack animals that are weak and sick. The parasites may be there at all times, but they do not attack the plants or animals successfully until the vitality is low. Wherever you find animal life you will find it has its parasites, and the most successful way to combat them is, if possible, to get within so much life, so much vigor, that they will be unable to get a foothold.

In my own household I do not feel it necessary to use the precautions which the doctor mentions, as my water-supply comes from a point sixty-nine feet below the surface and through thirty-five feet of dense hard pan, amounting almost to slate. With regard to the milk-supply, like our friend, I keep a cow in my stable, and I try to keep her clean enough so that she might be brought into the sitting-room if it were ever necessary.

*Dr. G. T. Baker.*—If I understood Dr. Hildreth, he stated that after forty years of age a man was practically immune from attacks of typhoid fever; how is it with extreme youth, or babies, especially those that are bottle-fed altogether?

*Dr. Hildreth.*—It is not known exactly at what age they become susceptible to the disease, but I think it is very probable that even very young babies, say two or three months old, are sometimes attacked by it. The cases are not very common until about five years old,—that is to say, the hospital records do not show anything much under that age, but I sometimes think that many cases are attended at home and may not be known as typhoid. I saw a case this summer of a child not more than six months old who had been sick with a disease which ran a course much the same as typhoid. The cases are comparatively rare under four and after forty, although I have known of a case of a man dying at sixty-five, and the autopsy showed the cause to have been typhoid fever. The most susceptible age is from twelve to twenty-five.

*Dr. Briggs.*—If the danger to milk is from handling, why is not there the same danger in handling of waters, as in bottling or barrelling? why is not there just as much danger of it by a man suffering from walking typhoid?

*Dr. Wilson.*—I would make a motion that a vote of thanks be extended to Dr. Hildreth for the very interesting and instructive talk he has given us this evening.

Unanimous vote.

William H. Potter, D.M.D.,
*Editor American Academy Dental Science.*

